# Death of Dr. Blythe

**Published:** 1842-06

**Authors:** 


### DEATH OF DR. BLYTHE.

Died at South Hanover, Indiana, on the 20th inst. (May), JAMES BLYTHE, D. D., first professor of Chemistry in the Medical Department of Transylvania University.

Dr. Blythe was a native of North Carolina, whence he emigrated to the State of Kentucky, in early life, leaving it about 10 years ago to accept the presidency of South Hanover College, still keeping himself in sight of the green hills of the State with which he had been so long identified. At the time of his death he was about 76 years of age; and had resigned the presidency several years before.

At an early period of life, almost in youth, Dr. Blythe was ordained as a minister of the Presbyterian church, and continued in that capacity till his death. Many years since he was appointed a professor in the Academical Department of the Transylvania University, in the establishment of which, indeed, he bore a conspicuous and important part. He afterwards resigned, and opened an academy for young ladies in Lexington, still, however, continuing an exemplary and solemn minister of the gospel.

In the year 1816, when the Trustees of Transylvania, under the special guidance of Dr. Dudley and Dr. Richardson, undertook the pioneer enterprise of establishing a Medical school in the West, Dr. Blythe was offered and accepted the chair of Chemistry. The faculty was organized in the autumn of 1817, when, from being one of its members, our acquaintance with the deceased commenced. The class of that, the first medical session ever held in the valley of the Mississippi, consisted of 20 pupils, and in the following March, 1818, a public commencement was held, and the degree of Doctor of Medicine conferred on one candidate. Thus Dr. Blythe's name is subscribed to the first diploma ever granted in the West, a *document* which our brethren of Transylvania should, if possible, have deposited in the archives of their *ancient*, and still prosperous university. The graduate was Dr. Lawson, a native of Lexington. After this commencement, we dissolved our connexion with the department, and, in the following summer, Dr. Blythe also resigned.—In the winter of 1818–19 no session was held, but in the ensuing autumn, two new professors (Drs. Brown and Caldwell) were elected and Dr. Blythe was reinstated. The school then reopened, and in 1823 we were elected a second time, and continued in it through four sessions, during which period we became intimately acquainted with the deceased, for whom we cherished a high personal respect. In 1831, 15 years after his original appointment, he took a final leave of the department, and was succeded by Dr. Yandell of Tennessee; public opinion seeming to require that the professor of Chemistry should be a medical man.

The members of the first faculty, appointed in 1816, were Drs. Dudley, Richardson, Overton, Blythe, and Drake, of whom all are still living except Dr. Blythe. Of the faculty of 1819, Dr. Brown died several years since. At a later period Dr. Eberle died, but as he entered the school in bad health and lectured but a few weeks, he should scarcely be ranked with its professors; so that we may say that from its foundation a quarter of a century ago, but two of its professors have died. Within that period, the University of Pennsylvania has lost five, and the Medical College of Ohio four, including Dr. Eberle.

Dr. Blythe was not a working chemist and of course had no great skill as a manipulator; but he was a classical scholar and adequately acquainted with the general principles of the science, which he presented to his class in a perspicuous manner. In his moral and social character he was honest, upright, and dignified; proud but not arrogant, firm yet not obstinate—abounding in self respect, but at all times disposed to respect the rights and feelings of others. The largest class which Transylvania ever had, listened to his lectures; and the most flourishing sessions of South Hanover College were under his administration.

But a few weeks before his death, we paid him a visit, and found that from several *slight* attacks of cerebral (apoplectic?) disease, his memory had become somewhat impaired; though he was still able to speak with zest of the early days of our *alma mater*. In former times rather corpulent, we now found him reduced in flesh; and on the whole presenting in both mind and body, a degree of senility disproportionate to his years. Of the immediate circumstances of his death we are ignorant.

Thus has passed away one of the first pioneers of medical education in the valley of the Mississippi. Although 50 years of age when he entered on his duties, he lived to see six other schools organized in that valley, with an aggregate of 750 pupils; a progress which has probably not been surpassed in any other country.

